# Enduring impact of communication skills training: results of a 12-month follow-up

**DOI:** 10.1038/sj.bjc.6601309

**Published:** 2003-10-14

**Authors:** L Fallowfield, V Jenkins, V Farewell, I Solis-Trapala

**Affiliations:** 1 Cancer Research UK Psychosocial Oncology Group, Brighton and Sussex Medical School, University of Sussex, Falmer, BN1 9QG, UK; 2 MRC Biostatistics Unit, Institute of Public Health, Robinson Way, Cambridge, CB2 2SR, UK

**Keywords:** communication skills training

## Abstract

The efficacy of a communication skills training programme was shown through a randomised trial. Oncologists (*N*=160) from 34 cancer centres were allocated to written feedback plus course; course alone; written feedback alone or control. Each clinician had 6 – 10 interviews with patients videotaped at baseline and 3 months postintervention. Analysis of videotapes revealed improvements in the communication skills of clinicians randomised to training (*n*=80) compared with others (*n*=80). A 12-month follow-up assessment is reported here. Robust Poisson conditional analyses of counts of changes in communication behaviours revealed no demonstrable attrition in those who had shown improvement previously, including fewer leading questions, appropriate use of focused and open-ended questions and responses to patient cues. Additional skills, not apparent at 3 months, were now evident; the estimated effect sizes corresponded to 81% fewer interruptions (*P*=0.001) and increased summarising of information to 38% (*P*=0.038). However, expressions of empathy (54%, *P*=0.001) declined. The overall results show that 12 – 15 months postintervention, clinicians had integrated key communication skills into clinical practice and were applying others. This is the first RCT to show an enduring effect of communication skills training with transfer into the clinic.

Communication is a core clinical skill, but is one in which few oncologists have received much formal training (Fallowfield *et al*, 1998; Maguire, 1999). Effective communication during consultations is the major determinant of the accuracy and completeness of data collection, influencing both the range and number of symptoms elicited, thus permitting a more precise assessment of the efficacy of treatment (Fallowfield and Jenkins, 1999). There is compelling evidence that communication affects numerous other important and meaningful health outcomes, such as adherence to drug regimens and diets, pain control and improvements in physical, functional and psychological well-being (Ong *et al*, 1995; Stewart, 1996). Poor communication can leave patients uncertain about their diagnosis and prognosis, confused about the results of diagnostic tests, unsure about further management plans or the therapeutic intent of treatment (Bruera *et al*, 2001). We also know that communication difficulties can impede the recruitment of patients to clinical trials, and therefore delay the introduction of potentially beneficial new treatments into the clinic (Jenkins *et al*, 1999). Oncologists themselves recognise that they have received inadequate communication or management skills training, and these together contribute to their own stress, lack of job satisfaction and emotional burnout (Ramirez *et al*, 1995, 1996).

The need to improve training has been acknowledged worldwide and is reflected in calls from many national and international organisations (DOH, 2000). For example, in the UK the Cancer Plan stated: ‘There will be a new joint training across professions in communication skills. By 2002 it will be a pre-condition of qualification that they are able to demonstrate competence in communication with patients. Advanced communication skills training will form part of continuing professional development programmes’. Until recently, the research base providing evidence of the efficacy of training comprised mainly exhortation, or description about isolated techniques that had neither been properly evaluated nor based on solid educational research (Cegala and Lenzmeier Broz, 2002). A recent systematic review (Fellowes *et al*, 2003) extracted 2822 papers on communication skills training in cancer health professionals, but only two of these (Fallowfield *et al*, 2002) and (Wilkinson *et al*, 1999) assessing clinicians and nurses, respectively, met the criteria for inclusion, that is, had a randomised design and objective analyses of skills postcourse.

Furthermore, as others have commented, few empirical data are available from methodologically sound studies, demonstrating that communication skills training transfers into the clinic situation and is then maintained over time (Baile *et al*, 1999; Libert *et al*, 2001).

Our RCT, cited in the systematic review, showed that an intensive 3-day training course significantly altered and improved senior clinicians' communication skills with patients in their clinics 3 months postcourse (Fallowfield *et al*, 2002). The study employed a 2 × 2 factorial design, in which 160 doctors from 34 UK cancer centres were randomised to either comprehensive written feedback and course, course alone, written feedback or control. Each doctor had 6 – 10 interviews with consenting patients videotaped at two time points, preintervention and then at 3 months. Two median length tapes, at each time point, for each doctor were analysed utterance-by-utterance using the Medical Interaction Process System (MIPS) (Ford *et al*, 2000). The MIPS permits classification of all patient and clinician utterances into content categories, which are basically the topic under discussion (e.g. side effects or sociodemographic information) and modes of exchange describing the manner in which information is either sought or offered (e.g. closed or open questions or expressions of preference or empathy).

Prior to seeing the doctor, patients completed a preference for information questionnaire (Jenkins *et al*, 2001) and the GHQ12, a brief psychological screening questionnaire (Goldberg and Williams, 1988; Fallowfield *et al*, 2001). Following the consultation, a researcher conducted an exit interview with patients to determine recall and patients' satisfaction with the consultation (Shilling *et al*, 2003). Doctors completed visual analogue scales rating their perception of how much patients had understood, how distressed the patient appeared and how satisfied they were with the consultation. Many other data were collected and will be reported at a later date. The study had multiregional ethics committee approval and fully informed, written consent was obtained from each doctor and patient involved prior to randomisation.

## THE TRAINING MODEL

Participants randomised to the training intervention had all attended a 3-day residential course using a model reported previously (Fallowfield *et al*, 1998). This model developed specifically for oncologists was based on one used extensively in the US (Lipkin *et al*, 1995). Briefly, courses were learner- centred, incorporating cognitive, affective and behavioural components. Participants worked in small groups of 3 – 5 individuals, led by an experienced facilitator together with a core team of six patient simulators (actors) skilled in providing constructive feedback from role.

In addition to the residential training course, participants received a comprehensive written feedback pack, which included analysis of the doctor's communication skills displayed in all videotaped interviews as well as the in-depth MIPS analysis, a summary of patient satisfaction scores and comments made by patients following their interviews, a summary of the congruency of the doctor's own ratings of patient distress and understanding of information with their patients' self-reports and brief exit interviews with researchers. The pack also contained a glossary of communication skills comprising words and phrases to assist the doctors' understanding of their written feedback and that received in training; for example, definitions of leading or focused, open questions, etc. Suggestions as to how individuals might change some of the communication patterns observed in their videos were made in addition to reinforcement of the effective skills they used that had been valued by patients. Finally, all were provided with an annotated bibliography and reprints of key references about effective communication skills.

A typical interview filmed in each doctor's clinic during the baseline assessment (T1) was reviewed in depth at the start of the course to illustrate and to elaborate upon comments made in their written feedback.

Adult learners respond best to educational endeavours that permit them to determine their own learning goals and include topics relevant to their own daily experience and interests (Cross, 1981). Consequently, each doctor identified the communication problems most important to them and worked on ways of resolving at least one of these through role play with patient simulators followed by video review and group discussion. The courses were given accreditation of 20 Continuing Medical Education points (CMEs) from the Royal Colleges of Surgeons, Physicians and Radiologists.

The training course was rated very highly by all participants, who felt that it had significantly influenced their attitudes and behaviours in clinics towards the importance of communicating well. These perceived changes were confirmed by objective analysis of follow-up data collected at T2 – 3 months postcourse or 3 months after first assessment in the case of controls (Jenkins and Fallowfield, 2002).

The Poisson regression analysis of counts of communication behaviours showed that course attendance significantly improved the key outcomes. Briefly, the estimated effect sizes corresponded to higher rates of desirable behaviours in course attendees compared to controls for focused and open questions (27%, *P*=0.005), expressions of empathy (69%, *P*=0.003), appropriate responses to patients' cues (38%, *P*=0.026) and led to a 24% decrease in the use of leading questions (*P*=0.11) (Fallowfield *et al*, 2002).

These results were very encouraging as it was the first rigorous RCT that had demonstrated unequivocal transfer of skills into the clinic setting. However, these types of training initiatives are extremely resource intensive and there is a dearth of trained facilitators. Doctors must be prepared to commit 3 days to the course and such educational interventions are considered fairly expensive to run. To encourage the likelihood of the uptake of the training methods nationally, there was a need to check whether the improvements found were enduring, so further assessments were made 12 months later (T3), in other words, 15 months or more since attendance at the communication skills course.

## SUBJECTS AND METHODS FOR T3 FOLLOW-UP

At the T3 assessment, 74 of the original 80 doctors randomised to the training course arms of the study had another 6 – 10 interviews with patients videotaped and analysed (two doctors had emigrated, two were working in research laboratories and no longer seeing patients and two were on maternity leave). The sample comprised 55 male and 19 female clinicians; 48 of whom were consultants and 26 were senior specialist registrars in training.

The types of communication skills displayed at some interviews are context dependent, for example, the content of an interview with a patient with lung cancer receiving news that the tumour was progressing would be rather different than one with a woman attending a follow-up clinic 5 years after successful treatment for primary breast cancer. We therefore ensured that similar groups of patients were involved in the T2 and T3 consultations. There were no apparent differences in the primary characteristics of patients seen at 3 months and 12 months postcourse in terms of age range, sex, tumour site, stage of disease, psychological status and intention of treatment, nor did the mean length of consultations differ; 12.3 min at 3 months compared with 11.71 min at 12 months (Table 1

**Table 1:** Characteristics of patients in videotaped consultations used for MIPS analysis at T2 (3 months) and T3 (12 months post-T2 assessment)

**Characteristics**	**T2, *n*=148**	**T3, *n*=148**
Male	56 (38%)	41 (28%)
Female	92 (62%)	107 (72%)
⩽30 years	17 (11%)	9 (6%)
31 – 50 years	35 (24%)	30 (20%)
51 – 70 years	76 (51%)	80 (54%)
>70 years	20 (14%)	29 (20%)
		
*Aim of treatment*		
Curative	64 (43%)	64 (43%)
Palliative	60 (40%)	64 (43%)
Remission	14 (10%)	14 (10%)
Uncertain/missing	10 (7%)	6 (4%)
		
*Cancer site*		
Breast	43 (29%)	43 (29%)
GI/colorectal	32 (22%)	31 (21%)
Urological	14 (9.5%)	9 (6%)
Gynaecological	10 (7%)	20 (13.5%)
Haematological	8 (5%)	10 (7%)
Lung	8 (5%)	9 (6%)
Skin/muscular/skeletal	9 (6%)	14 (9.5%)
Other/unknown/benign	14 (9.5%)	6 (4%)
Head and neck	5 (3%)	4 (3%)
CNS	5 (3%)	2 (1%)
		
*Length of interview*		
Mean (s.d.)	12.3 min (7.6)	11.71 min (5.1)
95% CI	11.05 – 13.5	10.89 – 12.56
		
*GHQ12*		
Below threshold <4	97 (65.5%)	97 (65.5%)
Above threshold ⩾4	51 (34.5%)	51 (34.5%)

).

As with the earlier assessments, two experienced raters blinded to previous evaluations analysed two of each doctor's median length videotapes using the MIPS (Ford *et al*, 2000). All results were then analysed by two independent medical statisticians.

## STATISTICAL METHODS

The analysis of the 3-month data from this trial was based on a comparison of the intervention and control groups. However, after this time point all control clinicians were offered the possibility of course attendance. Thus, the analysis of maintenance of performance over a 12-month period had to be based on a comparison of results at the beginning and end of this period for the same set of clinicians. Consequently, the statistical methodology used for the analysis was based on a Poisson regression model for the counts of behaviours in the two T2 and two T3 visits for which the MIPS analysis was carried out. In this model, log[expected (count)]=Ai for T2 visits and log[expected (count)]=Ai+log(RR) for T3 visits. The Ai parameters define physician-specific baseline rates of a particular behaviour at T2 and the relative rate parameter, RR, represents the change in the rate of the behaviour between T2 and T3. If RR=1, then no change is present.

The estimation of RR was based on a conditional likelihood. This allows an estimation of RR while adjusting for physician differences in baseline or T2 counts, effectively making this a before and after analysis. The definition of the conditional likelihood depends on the Poisson assumption, which may be questioned because of the clustering of counts within visits. However, a robust estimate of the variance of the estimated RR parameter was used and this makes the estimation results less dependent on the Poisson assumption than would be the case otherwise.

For the analysis of appropriate responses to cues, which obviously depend on the number of cues given, a conditional likelihood analysis was performed based on a binomial regression model. In this case, the shift between T2 and T3 behaviour is represented by an odds ratio parameter.

## RESULTS

Table 2

**Table 2:** Summary based on data from 74 doctors of robust Poisson conditional likelihood analyses comparing T2 (3 month) to T3 (12 months post-T2 assessment)

**Behaviour**	**RR (95% CI)**	***P*-value**	
Fewer leading questions	0.89 (0.65, 1.24)	0.5	Maintenance of skill shown to be higher than that of controls at 3 months in RCT
			
More focused questions	0.97 (0.81, 1.15)	0.69	Maintenance of skill shown to be higher than that of controls at 3 months in RCT
			
More focused open questions	0.96 (0.83, 1.10)	0.56	Maintenance of skill shown to be higher than that of controls at 3 months in RCT
Appropriate response to patient led cues	a1.02 (0.67, 1.54)	0.93	Maintenance of skill shown to be higher than that of controls at 3 months in RCT
			
Expressions of empathy	0.46 (0.33, 0.65)	<0.001	Attrition of skill shown to have been higher than that of controls at 3 months in RCT
			
More summarising of information	1.38 (1.02, 1.87)	0.038	Improvement in skill not previously seen to be higher than that of controls at 3 months
			
Fewer interruptions of patient	0.19 (0.10, 0.35)	<0.001	Improvement in skill not previously seen to be higher than that of controls at 3 months
			
Checking understanding	1.21 (0.88, 1.67)	0.24	No change, that is, no difference in behaviour at any time point compared with controls

a A patient cue has to be given for the doctor to make a response, therefore the statistic reported here is OR (odds ratio) from conditional binomial analysis T2 – T3.

gives the results of conditional likelihood analyses of data from 74 doctors.

The significantly more effective ways of asking questions of patients that doctors in the course arms of the trial had displayed at 3 months compared with controls was maintained at 12 months; that is, no significant attrition of these improved behaviours was observed for focused, focused and open and leading questions. The mean number of counts for the different types of questions per patient per visit was 4.79, 6.47 and 1.22 at 3 months compared with 4.63, 6.2 and 1.09 at 12 months, respectively.

Doctors also continued to respond appropriately to patient-led cues, 58% at 3 months *vs* 54% at 12 months, and inappropriate responses fell from 8 to 4%. The mean number of expressions of empathy dropped from 3.22 to 1.49. No change was found in the counts of effective checking of patient understanding.

Two further key behaviours had improved, that is, had a significant change since the 3-month assessment; there was an increase in the summarising of information for patients from 1.76 to 2.43; this corresponds to a 38% difference (*P*=0.038), and the total number of interruptions declined from 32 to only six interruptions at 12 months, which corresponds to an 81% difference (*P*<0.001).

## DISCUSSION

Overall, these results demonstrate that approximately 15 months postcourse, and without any further training interventions, the doctors had integrated well many of the key communication skills that they had learned into their normal practice. They were also exhibiting additional, important and effective skills. There was no demonstrable attrition in all but one of the five desirable communication behaviours that had shown significant improvement at 3 months. Counts of leading questions, which result in faulty data collection, remained low. Doctors continued to employ focused and focused open questions, valuable skills, particularly at the beginning of an interview, to enable collection of more reliable information and to help identify patient concerns. Efficient questioning was accompanied by a decrease in the number of times that patients were interrupted, an extra skill that had not been apparent at 3 months. Adoption of a more patient-centred interviewing style enables doctors to elicit a broader perspective of how the disease and its treatment impacts upon patients. Listening without interruption facilitates understanding and promotes a better therapeutic relationship (Beckman and Frankel, 1984).

The increase in the summarising of information given to patients is also an important additional skill. Oncology consultations invariably contain large amounts of complex and potentially distressing information, so summarising is especially valuable when discussing a life-threatening disease, complicated management plans and the likely therapeutic benefits. However, no change was found in the counts of effective checking of patient understanding, a behaviour often omitted in busy clinics. A point worth emphasising is that the use of other effective communication skills did not increase the length of consultations.

The apparent attrition in counts of empathy since time point 2 was disappointing and needs to be addressed. The expression of empathy requires the doctor to possess first the skills to do it and then the ability to recognise when it is needed. Expressing empathy is also context dependent. We therefore checked whether or not the patient characteristics, in particular psychological status or treatment intent, differed between those seen at 3 and 12 months; none was observed. Empathy can be displayed in ways other than words. As the MIPS system does not measure nonverbal communication extensively, the videos of doctors who had shown the greatest apparent decline in empathy were reviewed again. It was clear that instead of interrupting patients during emotional statements, the doctors were exhibiting empathy nonverbally through nods, facial expressions and other gestures such as touching.

The maintenance of most of the desirable communication behaviours can also be interpreted as an increase in the doctors' sense of self-efficacy. According to Bandura (1977), individuals with strong beliefs in their ability to perform a behaviour successfully are more likely to initiate the behaviour and persist through difficulties. This theory has previously been applied to research on communication in oncology (Parle *et al*, 1997).

We know that our Cancer Research UK course significantly shifted doctors' attitudes and beliefs about the importance of communicating well compared with that of controls and increased the likelihood of them displaying important skills in clinics (Jenkins and Fallowfield, 2002). It is possible that as time went on, the doctors found that the use of their new skills led to benefits, which also reinforced a belief in their ability to perform the behaviour successfully. In other words, desirable communication behaviours were maintained because the outcome from using the new skills produced acceptable consequences; for example, using a focused open questioning style helped rather than hindered patient history taking. Bandura's theory could also explain why new behaviours emerged in our clinicians 12 months following the previous assessment. Once skills were regularly incorporated into the clinical routine and with an increased self-efficacy about their ability to employ these in the consultation, other more advanced behaviours could emerge.

These results, showing the enduring impact of a course designed to improve oncologists' communication skills, are an important contribution to the growing literature in this area as many have questioned whether or not behaviour changes are transferable and moreover sustainable in the clinic setting. Course methods that incorporate a learner-centred approach with cognitive, affective and behavioural components, which encourage constructive forms of self-critique, appear to enhance continued improvements in skills. Such courses are resource intensive and expensive, but are probably necessary if changes in both attitudes and beliefs as well as communication behaviours are to occur and if skills are transferred and maintained in a pressured clinical environment (Jenkins and Fallowfield, 2002).

The Department of Health in the UK has launched a nationwide communication skills training programme based on models similar to the one described here, through the new NHS University in collaboration with Cancer Research UK and Marie Curie Cancer Care. We hope that this short report demonstrating the enduring nature of effective communication skills programmes will help others worldwide trying to secure funding for better training for all healthcare professionals working within oncology.

## CONTRIBUTORS

Lesley Fallowfield conceived the original study, wrote the protocol, obtained funding and facilitated the training courses. Valerie Jenkins contributed to data collection, coordinated day-to-day management of the project, participated in the preparation of data analysis and coauthored the first drafts of the manuscript. Vern Farewell contributed to the study design, writing of the protocol and statistical analysis plan and with Ivonne Solis-Trapala analysed the 12-month follow-up results. All authors contributed to the writing of the final manuscript. Lesley Fallowfield is the guarantor for the report. None of the authors have any conflicts of interest.

## References

[bib1] Baile W, Kudelka AP, Beale EA, Glober GA, Myers MA, Greisinger AJ, Bast RC, Goldstein MG, Novack D, Lenzi R (1999) Communication skills training in oncology. Cancer 86: 887–897 10463990

[bib2] Bandura A (1977) Self-efficacy: toward a unifying theory of behavioral change. Psychol Rev 84: 191–215 847061 10.1037//0033-295x.84.2.191

[bib3] Beckman HB, Frankel RM (1984) The effect of physician behavior on the collection of data. Ann Intern Med 101: 692–696 6486600 10.7326/0003-4819-101-5-692

[bib4] Bruera E, Sweeney C, Calder K, Palmer L, Benisch-Tolley S (2001) Patient preferences versus physician perceptions of treatment decisions in cancer care. J Clin Oncol 19: 2883–2885 11387361 10.1200/JCO.2001.19.11.2883

[bib5] Cegala DJ, Lenzmeier Broz S (2002) Physician communication skills training: a review of theoretical backgrounds, objectives and skills. Med Educ 36: 1004–1016 12406260 10.1046/j.1365-2923.2002.01331.x

[bib6] Cross KP (1981) Adults As Learners. San Francisco: Jossey-Bass

[bib7] DOH (2000) The NHS Cancer Plan, pp 1–97. London: Department of Health

[bib8] Fallowfield L, Jenkins V, Farewell V, Saul J, Duffy A, Eves B (2002) Efficacy of a Cancer Research UK communication skills model: a randomised controlled trial. Lancet 359: 650–656 11879860 10.1016/S0140-6736(02)07810-8

[bib9] Fallowfield L, Ratcliffe D, Jenkins V, Saul J (2001) Psychological morbidity and its recognition by doctors in patients with cancer. Br J Cancer 84: 1011–1015 11308246 10.1054/bjoc.2001.1724 PMC2363864

[bib10] Fallowfield LJ, Jenkins VA (1999) Effective communication skills are the key to good cancer care. Eur J Cancer 35: 1592–1597 10673967 10.1016/s0959-8049(99)00212-9

[bib11] Fallowfield LJ, Lipkin M, Hall A (1998) Teaching senior oncologists communication skills: results from phase 1 of a comprehensive longitudinal program in the UK. J Clin Oncol 16: 1961–1968 9586916 10.1200/JCO.1998.16.5.1961

[bib12] Fellowes D, Wilkinson S, Moore P (2003) Communication skills training for health care professionals working with cancer patients, their families and/or carers. Cochrane Database Syst Rev CD003751 10.1002/14651858.CD003751 12804489

[bib13] Ford S, Hall A, Ratcliffe D, Fallowfield L (2000) The Medical Interaction Process System (MIPS): an instrument for analysing interviews of oncologists and patients with cancer. Soc Sci Med 50: 553–566 10641807 10.1016/s0277-9536(99)00308-1

[bib14] Goldberg D, Williams P (1988) A User's Guide to the General Health Questionnaire. Windsor: NFER-Nelson

[bib15] Jenkins V, Fallowfield L (2002) Can communication skills training alter physicians? beliefs and behavior in clinics? J Clin Oncol 20: 765–769 11821459 10.1200/JCO.2002.20.3.765

[bib16] Jenkins V, Fallowfield L, Saul J (2001) Information needs of patients with cancer: results from a large study in UK cancer centres. Br J Cancer 84: 48–51 11139312 10.1054/bjoc.2000.1573 PMC2363610

[bib17] Jenkins VA, Fallowfield LJ, Souhami A, Sawtell M (1999) How do doctors explain RCTs to their patients. Eur J Cancer 35: 1187–1193 10615228 10.1016/s0959-8049(99)00116-1

[bib18] Libert Y, Conradt S, Reynaert C, Janne P, Tordeurs D, Delvaux N, Fontaine O, Razavi D (2001) Improving doctor's communication skills in oncology: review and future perspectives. Bull Cancer 88: 1167–1176 11792610

[bib19] Lipkin M, Kaplan C, Clark W, Novack D (1995) Teaching medical interviewing: the Lipkin model. In The Medical Interview. Clinical Care Education, and Research, Lipkin M, Putnam SM, Lazare A (eds) pp 422–435. New York: Springer-Verlag

[bib20] Maguire P (1999) Improving communication with cancer patients. Eur J Cancer 35: 2058–2065 10711246 10.1016/s0959-8049(99)00301-9

[bib21] Ong LM, de Haes JC, Hoos AM, Lammes FB (1995) Doctor – patient communication: a review of the literature. Soc Sci Med 40: 903–918 7792630 10.1016/0277-9536(94)00155-m

[bib22] Parle M, Maguire P, Heaven C (1997) The development of a training model to improve health professionals' skills, self-efficacy and outcome expectancies when communicating with cancer patients. Soc Sci Med 44: 231–240 9015875 10.1016/s0277-9536(96)00148-7

[bib23] Ramirez AJ, Graham J, Richards MA, Cull A, Gregory WM (1996) Mental health of hospital consultants: the effects of stress and satisfaction at work. Lancet 347: 724–728 8602002 10.1016/s0140-6736(96)90077-x

[bib24] Ramirez AJ, Graham J, Richards MA, Cull A, Gregory WM, Leaning MS, Snashall DC, Timothy AR (1995) Burnout and psychiatric disorder among cancer clinicians. Br J Cancer 71: 1263–1269 7540037 10.1038/bjc.1995.244 PMC2033827

[bib25] Shilling V, Jenkins V, Fallowfield L (2003) Factors affecting patient and clinician satisfaction with the clinical consultation: can communication skills training for clinicians improve satisfaction? Psycho-Oncology 12: 599–611 12923800 10.1002/pon.731

[bib26] Stewart MA (1996) Effective physician – patient communication and health outcomes: a review. Can Med Assoc J 152: 1423–1433 PMC1337906 7728691

[bib27] Wilkinson S, Bailey K, Aldridge J, Roberts A (1999) A longitudinal evaluation of a communication skills programme. Palliat Med 13: 341–348 10659103 10.1191/026921699672159169

